# A Metabolic Study on Colon Cancer Using ^1^H Nuclear Magnetic Resonance Spectroscopy

**DOI:** 10.1155/2014/348712

**Published:** 2014-08-14

**Authors:** Zahra Zamani, Mohammad Arjmand, Farideh Vahabi, Seyed Mahmood Eshaq Hosseini, Sadegh Mohammad Fazeli, Ayda Iravani, Parastoo Bayat, Akbar Oghalayee, Mahshid Mehrabanfar, Reza Haj Hosseini, Mohammad Tashakorpour, Mohsen Tafazzoli, Sedigheh Sadeghi

**Affiliations:** ^1^Biochemistry Department, Pasteur Institute of Iran, Tehran 1316943551, Iran; ^2^Amir Alam Hospital, North Sa'adi Avenue, Tehran 8915964665, Iran; ^3^Imam Khomeini Hospital, Dr. Gharib Road, Tehran 1419733141, Iran; ^4^Biotechnology Department, Pasteur Institute of Iran, Tehran 1316943551, Iran; ^5^Payame Noor University, Tehran 19569, Iran; ^6^Sharif University, Azadi Avenue, Tehran 11559567, Iran

## Abstract

*Background*. Colorectal carcinoma is the third cause of cancer deaths in the world. For diagnosis, invasive methods like colonoscopy and sigmoidoscopy are used, and noninvasive screening tests are not very accurate. We decided to study the potential of ^1^HNMR spectroscopy with metabolomics and chemometrics as a preliminary noninvasive test. We obtained a distinguishing pattern of metabolites and metabolic pathways between colon cancer patient and normal. *Methods*. Sera were obtained from confirmed colon cancer patients and the same number of healthy controls. Samples were sent for ^1^HNMR spectroscopy and analysis was carried out Chenomex and MATLAB software. Metabolites were identified using Human Metabolic Data Base (HDMB) and the main metabolic cycles were identified using Metaboanalyst software. *Results*. 15 metabolites were identified such as pyridoxine, orotidine, and taurocholic acid. Main metabolic cycles involved were the bile acid biosynthesis, vitamin B6 metabolism, methane metabolism, and glutathione metabolism. *Discussion*. The main detected metabolic cycles were also reported earlier in different cancers. Our observations corroborated earlier studies that suggest the importance of lowering serum LCA/DCA and increasing vitamin B6 intake to help prevent colon cancer. This work can be looked upon as a preliminary step in using ^1^HNMR analysis as a screening test before invasive procedures.

## 1. Introduction

Colorectal carcinoma (CRC) ranked third as the cause of cancer death in the world. It is estimated that 142,820 people will be diagnosed with CRC and 50,830 men and women will die of it in 2014. In the US the death rate has dropped due to screening and the age-adjusted incidence rate is 45.0 per 100,000 [1]. The mortality rate in the US has dipped sharply due to public awareness and insurance support for screening tests after the age of fifty. In Iran the case of CRC is on the rise from 6 to 7.9 per 100,000 in 2005 to 38.0 per 100,000 in 2012 and is the fourth common cancer [2].

CRC screening is carried out by different procedures such as fecal occult blood test (FOBT), sigmoidoscopy, colonoscopy, virtual colonoscopy, and double contrast barium enema (DCBE). Each has its own advantages and disadvantages. A digital rectal exam (DRE) during routine physical examinations is performed by some physicians and they may use this procedure to check the lower part of the rectum [3].

Noninvasive methods do not seem to be very accurate but are more economical and easier to perform. There are two FOBT tests to detect the presence of hemoglobin in stool. One uses a dye for detection, guaiac FOBT, and the other fecal immunochemical testing uses specific immunoglobulin. This method is performed every two or three years for individuals above fifty years and is effective in reducing the cases of CRC by 15 to 33 percent [4]. A digital rectal exam and contrast barium enema are also used for screening but cannot detect about 50% of polyps identified by colonoscopy [5]. Sigmoidoscopy and colonoscopy are invasive but seem to be the most effective tools of diagnosis of CRC [6, 7]. A combination of methods used depends on many factors such as patient history, age, and insurance coverage in many countries [8].

The genetic changes in CRC have been studied extensively. Metabolomics represents one of the new omics sciences which takes advantage of the unique presence and concentration of small molecules in tissues and body fluids to make a fingerprint that can be unique to the individual. Metabolomics has the potential to serve an important role in diagnosis and management of many human disorders such as CRC. More investigations are required as small molecules like the metabolites are difficult to characterize and require high throughput technology [9]. Recently with the advent of new technology like mass spectrometry and ^1^H nuclear magnetic resonance (^1^HNMR), the provision for analyzing small molecules are carried out [10]. After obtaining the spectral pattern for the required samples, chemometrics is done using different mathematical modeling like principle component analysis (PCA), partial linear square (PLS), or PLS-DA (discriminate analysis) [11, 12]. This gives us a pattern to distinguish the metabolites between normal and abnormal samples. Using the pattern obtained by the differentiating chemical shifts and the Human Metabolome Database (HDMB), the metabolites are identified. Using other software like Metaboanalyst and KEGG pathway analysis, the main pathways involved are obtained [13].

Since most CRC screening methods such as colonoscopy and sigmoidoscopy are invasive, we decided to use high throughput technology and find out a metabolite pattern for cancer patients which would differentiate them from normal.

## 2. Materials and Methods

### 2.1. Sample Collection

5 mL of blood was collected from people who were on a liquid diet for at least 48 hours and were referred for colonoscopy to the Gastroenterology Department at Amir Alam Hospital, Tehran. One group comprised of 33 patients diagnosed with cancer by colonoscopy and biopsy and the second group of individuals without colon cancer. Sera were separated and stored at −80°C. All groups were made to fill a consent form as per the requirements of Pasteur Institute Ethics Committee before serum collection.

### 2.2. Sample Preparation for ^1^HNMR Spectroscopy

600 *μ*L of serum with 70 *μ*L D_2_O and 1 mM sodium 2-trimethylsilylpropionate (TMSP) was used as internal reference in a 5 mm NMR tube at room temperature and data acquisition was carried out.

For NMR data collection, one-dimensional ^1^HNMR spectra were acquired on a Bruker DRX-500 NMR spectrometer operating at 500.13 MHZ and Carr-Purcell-Meiboom-Gill (CPMG) 90-(t-180-tn-acqusition) (*τ* = 200, *n* = 100) pulse sequence as described earlier.

Analysis of data and pattern recognition were performed using Chenomix 6.4 software.

### 2.3. Chemometrics Analysis

#### 2.3.1. Principle Component Analysis (PCA)

Initially the NMR variables were mean centered; then PCA was used to detect the outliers data [11] for detecting strong outliers and by Q residuals for detecting modest outliers with 95% confidence level.

#### 2.3.2. Partial Linear Square (PLS)

PLS is a supervised method that uses multivariate regression technique to extract via linear combination of original variables (**X**) the information that can predict the class membership (**Y**). PLS was applied after OSC using the** Y** matrix including 0 for normal and 1 for abnormal for all the data set. PLS was performed with and without OSC and results were obtained with more than 95% confidence [12].

OSC filters were developed to remove unwanted variation from spectral data. PLS was applied after OSC using the** Y** matrix including 0 for normal and 1 for abnormal for all the data set described formerly [14]. Matrix** X** comprises of ^1^HNMR data of samples from normals and matrix** Y** samples from cancer patients. OSC subtracts from** X**, factors that account for as much as possible the variance in** X** and are orthogonal to** Y**. It is important to avoid overfitting after OSC treatment, so as to prevent poor predictive performance. Hence, precise determination of the number of removed OSC factors is very important. Only one factor was removed.

#### 2.3.3. Human Metabolome Database (HDMB)

The NMR search link of HDMB was used to detect the metabolites of certain chemical shifts which contains information about metabolites found in the human body [15].

#### 2.3.4. Metabolic Pathway Analysis

It was performed with Metaboanalyst 2.0 for pathway analysis and visualization. These pathways were affected by the metabolites in colon cancer patients.

## 3. Results

**Figure 1:**
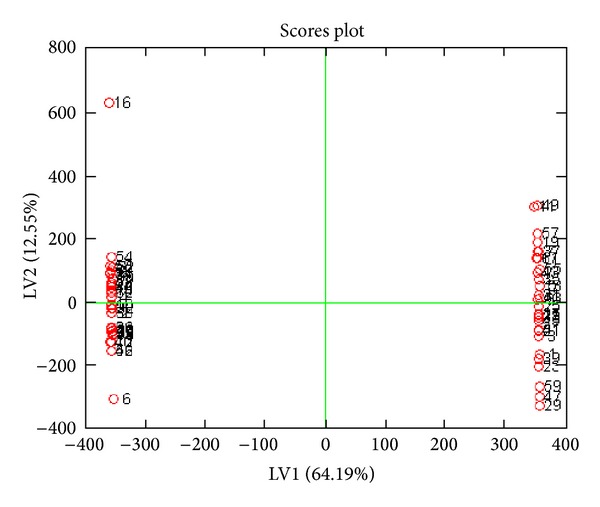
Score plot of PLS after OSC shows very good separation of samples. Odd numbers indicate normal and even numbers patient samples.

**Figure 2:**
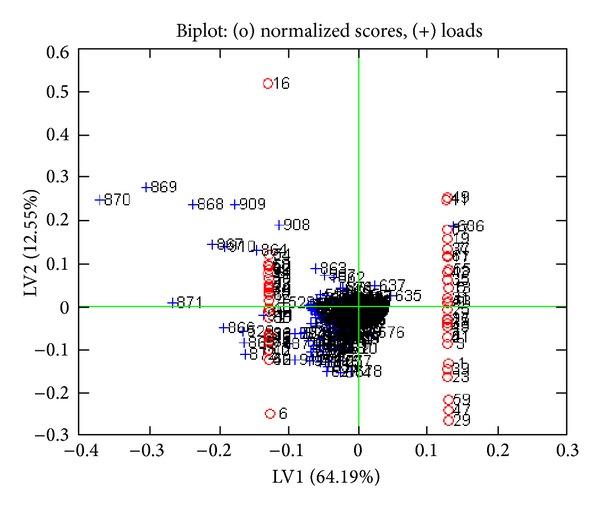
Biplot of PLS after OSC showing differentiating metabolites. Odd numbers indicate normal and even number of patient samples.

**Figure 3:**
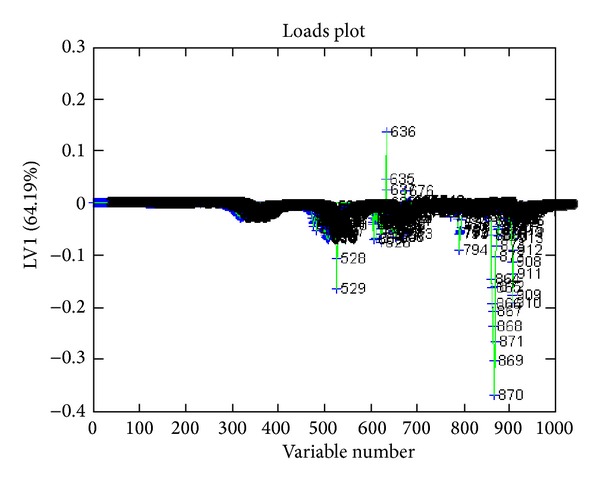
Loads plot of metabolites below the axis show descending levels and above the axis show ascending levels of metabolites. Numbers indicate metabolites.

**Figure 4:**
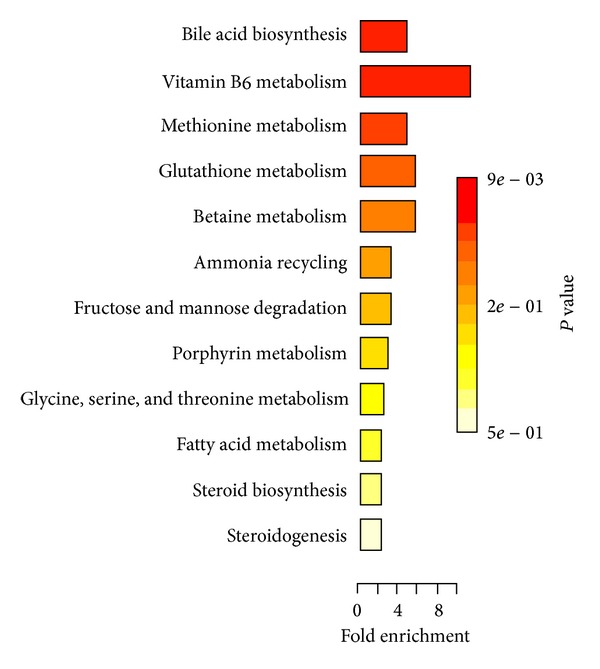
Summary plot for over representation analysis (ORA).

PLS was applied after OSC using the** Y** matrix including 0 for normal and 1 for abnormal for all the data set. PLS was performed with and without OSC and results were obtained with more than 95% confidence level. Figures 1 and 2 show a complete separation pattern between the colon cancer and the normal groups.  Figure 3 shows loading plot of the samples which is an indicator of ascending and descending level of metabolites. With the help of the numbers and chemical shifts, 13 metabolites were identified as shown in Table 1. 15 metabolic pathways were detected from the above-differentiating metabolites after an enrichment analysis was carried out in Figure 4. Overrepresentation analysis, as shown in Table 2, was done to detect the impact of pathways, depending on the number of changed metabolites and to test if a particular group of compounds is represented more than expected by chance within the user uploaded compound list in the Metaboanalyst software. In the context of pathway analysis, compounds involved in a particular pathway are enriched and compared by random hits as tested. The detailed results from the pathway analysis are depicted in Table 2, and, since many pathways are tested at the same time, the statistical *P* values from enrichment analysis are further adjusted for multiple tests.

## 4. Discussion

In this study a number of metabolites and their pathways were detected which have an important impact on colon cancer. Of all the cycles involved, primary bile acid biosynthesis and degradation of ketone bodies and cyanoamino acid metabolism were the major ones (Table 2). Of all the metabolites involved, the main ones were cholesterol, glycine, glycocholic acid, and taurocholic acid which are involved in primary bile acid biosynthesis. Secondary bile acids are formed by enzymatic deconjugation and dehydroxylation of the primary bile acids by anaerobic bacteria in the large intestine. Reports show the presence of higher deoxycholic acids in serum and bile of the patients with colonic adenomas than in the healthy controls [16]. Studies have shown that these secondary bile acids exhibit tumour-promoting ability in animals and they could trigger apoptosis and may also act as regulatory molecules involved in different cell signaling pathways in colon cells [17]. It is interesting that the ratio of LCA (lithocholic acid)/DCA (deoxycholic acid) may be an important factor to distinguish tendency to colon cancer [18]. Reduction of LCA/DCA is reported on experiments carried out on rats fed on a high diet supplemented with vitamin B6 [19]. The second metabolic cycle involved in colon cancer is vitamin B6. There are several theories for the role of this vitamin. It upregulates a protective factor, insulin like growth factor binding protein 1 (IGFBP1); its mRNA is upregulated in HT29 colon carcinoma cells exposed to pyridoxal (a form of vitamin B6). IGFBP1 is secreted from the liver and is hypothesized to exert a protective role in the development of cancer and cardiovascular diseases [20]. ELISA indicated analysis showed that supplemental vitamin B6 significantly lowered levels of colonic HSP70, heme-oxygenase-1, and HSP32 which increase cell proliferation and colonic damage. Heat shock proteins (HSPs, molecular chaperones) have been suggested to be associated with colon carcinogenesis [21]. In mice receiving the colonic carcinogen azoxymethane, the development of colonic aberrant crypt foci, precursor lesions of colon cancer, and cell proliferation are suppressed by vitamin B6 supplementation [22].

The next effective cycle is the synthesis and degradation of ketone bodies with 3-hydroxybutyric acid as the metabolite involved. It is interesting to note that recent studies suggest that a ketogenic diet helps overcome different kinds of cancers. This is attributed to the fact that most malignant cells depend on glucose as fuel and cannot metabolize fatty acids easily due to dysfunction of the mitochondria. Malignant cells grown* in vitro* are negatively affected by low glucose and a similar antitumorigenic property of low carbohydrate diets is shown in mice* in vivo* experiments [23]. 3-Hydroxybutyric acid is also a biomarker detected by GC-MS from sera of colorectal patients [24].

Glycine is a very important metabolite as it takes part in the next four cycles of cyanoamino metabolism, thiamine metabolism, methane metabolism, and glutathione metabolism. Association between increase in serum glycine and colon cancer has been shown in many studies [25]. Glycine is differentiating metabolites for colon cancer as seen in metabolomic studies by time of flight mass spectrometry (TOFMS) [26]. An* in vitro* study carried out on cancer cells by metabolite profiling indicates key role of glycine in cancer cell proliferation [27].

Thiamine or vitamin B1 has an important role in cancer cells as shown by investigations using the thiamine-degrading enzyme, thiaminase. Liu et al. showed that the addition of thiaminase into cell culture media containing thiamine had a significant inhibitory effect on growth of breast cancer cells [28]. Also a pegylated version of thiaminase was capable in delaying tumor growth and prolonging survival in an RS4 leukemia xenograft model [29, 30] and it is seen that the increased glycine and thiamine are linked.

Methane metabolism by bacteria in the large intestine has been reported as early as 1977. This report showed that excretion of methane in breath occurred twice as frequent in patients with colonic cancer as normal individuals [31]. This suggests the difference between the anaerobic intestinal flora in patients and normal subjects and implies that colorectal cancer may be caused by carcinogens formed by nuclear bile acid dehydrogenation in the large intestine by anaerobic bacteria [32]. The glutathione pathway is seen after increasing glycine. Glutathione levels in primary colorectal cancer tissues were significantly higher than in the corresponding normal tissues. Reports show that elevated glutathione levels had a significant negative effect on survival rate in patients with colorectal cancer [33].

Reports have shown that in colon cancer the metabolism and catabolism of amino acid increase. Glycine also is seen to participate in glycine, serine, and threonine metabolism which increases in colon cancer and is part of nitrogen metabolism [34].

(R)-3-Hydroxybutyric acid is very important in butanoate metabolism. The antitumor effects of butyrate were described in studies using colorectal cancer cell lines in which butyrate inhibits growth and induces differentiation and apoptosis [35]. In other studies butyrate was able to inhibit tumor growth* in vivo* in murine models [36]. But there are conflicting reports about the protective role of butyrate as seen that colorectal cells still increase and grow even though there are high concentrations of butyrate in the colon [37].

Fucose and mannose metabolism are affected by L-fucose which is an important posttranslational modification in cancer and inflammation. Sera and total cellular proteins of cancer patients showed increase in fucosylation levels and recently some fucosylated proteins have been identified as novel cancer biomarkers in glycoproteomic analyses [38].

## 5. Conclusions

Using ^1^HNMR analysis and chemometrics, a differentiation pattern was obtained between the metabolites in the sera of colon cancer patients and normals. Using HDMB 15 main metabolites were identified and Metaboanalyst software detected 13 metabolic cycles which had been reported as playing an important part in cancers and tumor progression. The main pathways were bile acid biosynthesis and vitamin B6 biosynthesis, and our study corroborates early findings and suggests the importance of lowering serum LCA/DCA and increasing vitamin B6 intake to help prevention of colon cancer.

## Figures and Tables

**Table 1:** Differentiating metabolites between cancer and patient groups.

Number of metabolite	Name of metabolite in serum	HDMB number	Level in serum
1	Pyridoxine	HMDB00239	↓
2	Orotidine	HMDB00788	↓
3	S-adenosylhomocysteine	HMDB00939	↓
4	Pyridoxamine	HMDB01431	↓
5	Glycocholic acid	HMDB00138	↓
6	Beta-leucine	HMDB03640	↓
7	5-Methylcytidine	HMDB00982	↓
8	Taurocholic acid	HMDB00036	↓
9	3-Hydroxybutyric acid	HMDB00357	↓
10	7-Ketocholesterol	HMDB00501	↓
11	3-Hydroxyisovaleric acid	HMDB00754	↓
12	L-fucose	HMDB00174	↓
13	Cholesterol	HMDB00067	↓
14	L-palmitoylcarnitine	HMDB00222	↓
15	Glycine	HMDB00123	↑

Differentiating metabolites detected from their chemical shifts and identified by HDMB. Ascending and descending levels of metabolites shown in the sera with arrows.

**Table 2:** Result from pathway analysis.

	Total	Expected	Hits	Raw *P*
Primary bile acid biosynthesis	47	0.23	4	5.64*E* − 05
Vitamin B6 metabolism	32	0.16	2	1.04*E* − 02
Synthesis and degradation of ketone bodies	6	0.03	1	2.96*E* − 02
Cyanoamino acid metabolism	16	0.08	1	7.71*E* − 02
Taurine and hypotaurine metabolism	20	0.10	1	9.55*E* − 02
Thiamine metabolism	24	0.12	1	1.14*E* − 01
Methane metabolism	34	0.17	1	1.57*E* − 01
Glutathione metabolism	38	0.19	1	1.74*E* − 01
Nitrogen metabolism	39	0.19	1	1.78*E* − 01
Butanoate metabolism	40	0.20	1	1.83*E* − 01
Valine, leucine and isoleucine degradation	40	0.20	1	1.83*E* − 01
Lysine degradation	47	0.23	1	2.11*E* − 01
Fructose and mannose metabolism	48	0.24	1	2.15*E* − 01
Glycine, serine, and threonine metabolism	48	0.24	1	2.15*E* − 01
Fatty acid metabolism	50	0.25	1	2.23*E* − 01
Cysteine and methionine metabolism	56	0.28	1	2.47*E* − 01
Pyrimidine metabolism	60	0.30	1	2.62*E* − 01
Aminoacyl-tRNA biosynthesis	75	0.37	1	3.17*E* − 01
Amino sugar and nucleotide sugar metabolism	88	0.44	1	361*E* − 01
Purine metabolism	92	0.46	1	3.74*E* − 01
Steroid hormone biosynthesis	99	0.49	1	3.97*E* − 01
Porphyrin and chlorophyll metabolism	104	0.52	1	4.12*E* − 01

The Total is the total number of compounds in the pathway and the Hits are actually matched number from the user uploaded data. The Raw *P* is the original *P* value calculated from the enrichment analysis.
